# 4-Nitro-*N*-(4-pyridinio)benzene­sulfonamidate monohydrate

**DOI:** 10.1107/S1600536808032273

**Published:** 2008-10-11

**Authors:** Yu-Zhen Chen, Han Gao, Gang Li, Xiao-Jing Chen, Sheng-Yang Niu

**Affiliations:** a Department of Mathematics, Henan Institute of Science and Technology, Xinxiang 453003, People’s Republic of China; b School of Food Science, Henan Institute of Science and Technology, Xinxiang 453003, People’s Republic of China; c Xinke College, Henan Institute of Science and Technology, Xinxiang 453003, People’s Republic of China

## Abstract

The title compound, C_11_H_9_N_3_O_4_S·H_2_O, contains both an acid and a base centre, and displays a zwitterionic structure in the solid state. The benzene ring makes an angle of 109.1 (2)° with the pyridinium ring. The crystal structure is stabilized by O—H⋯N, O—H⋯O and N—H⋯O hydrogen bonds.

## Related literature

For related literature, see: Allen *et al.* (1987 ▶); Li *et al.* (2007 ▶); Damiano *et al.* (2007 ▶); Yu & Li (2007 ▶).
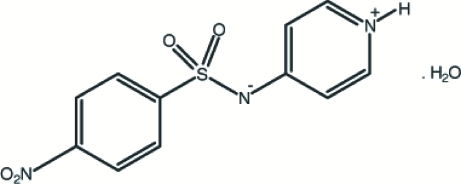


## Experimental

### 

#### Crystal data

C_11_H_9_N_3_O_4_S·H_2_O
*M* *_r_* = 297.29
Monoclinic, 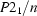

*a* = 6.7766 (14) Å
*b* = 8.3932 (17) Å
*c* = 21.717 (4) Å
β = 92.35 (3)°
*V* = 1234.2 (4) Å^3^
*Z* = 4
Mo *K*α radiation
μ = 0.29 mm^−1^
*T* = 113 (2) K
0.20 × 0.18 × 0.12 mm

#### Data collection

Rigaku Saturn CCD area-detector diffractometer
Absorption correction: multi-scan (*CrystalClear*; Rigaku/MSC, 2005 ▶) *T*_min_ = 0.932, *T*_max_ = 0.963
9708 measured reflections
2908 independent reflections
2022 reflections with *I* > 2σ(*I*)
*R*_int_ = 0.061

#### Refinement

*R*[*F* ^2^ > 2σ(*F* ^2^)] = 0.055
*wR*(*F* ^2^) = 0.136
*S* = 1.17
2908 reflections
193 parameters
H atoms treated by a mixture of independent and constrained refinement
Δρ_max_ = 0.35 e Å^−3^
Δρ_min_ = −0.45 e Å^−3^

### 

Data collection: *CrystalClear* (Rigaku/MSC, 2005 ▶); cell refinement: *CrystalClear*; data reduction: *CrystalClear*; program(s) used to solve structure: *SHELXS97* (Sheldrick, 2008 ▶); program(s) used to refine structure: *SHELXL97* (Sheldrick, 2008 ▶); molecular graphics: *SHELXTL* (Sheldrick, 2008 ▶); software used to prepare material for publication: *SHELXTL*.

## Supplementary Material

Crystal structure: contains datablocks global, I. DOI: 10.1107/S1600536808032273/at2641sup1.cif

Structure factors: contains datablocks I. DOI: 10.1107/S1600536808032273/at2641Isup2.hkl

Additional supplementary materials:  crystallographic information; 3D view; checkCIF report

## Figures and Tables

**Table 1:** Hydrogen-bond geometry (Å, °)

*D*—H⋯*A*	*D*—H	H⋯*A*	*D*⋯*A*	*D*—H⋯*A*
O5—H5*A*⋯N2	0.85 (4)	1.97 (4)	2.813 (3)	167 (4)
O5—H5*B*⋯O2^i^	0.85 (5)	2.07 (5)	2.895 (3)	164 (4)
N1—H1*A*⋯O5^ii^	0.92 (4)	1.82 (4)	2.728 (3)	170 (4)

Symmetry codes: (i) 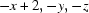; (ii) 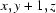.

**Fig. 1.**
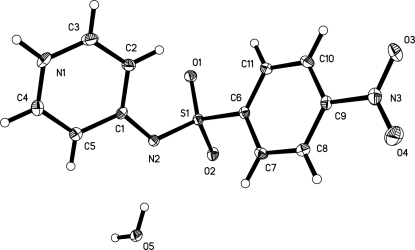
Molecular view of the title compound (I) showing the atom-labelling scheme. Displacement ellipsoids are drawn at the 35% probability level (arbitrary spheres for the H atoms).

## supplementary crystallographic information

### Comment

Organic pyridinium salts have been widely used in the construction of supramolecular architectures (Teresa *et al.*, 2007). As part of our ongoing studies of supramolecular chemistry involving the pyridinium rings (Li *et al.*, 2007), the structure of the title compound (I) was determined by X-ray diffraction.

In the cations of (I) (Fig. 1), the short C—N distance [N2—C1 = 1.370 (3) Å] has a value between those of a typical C═N double and C—N single bond [1.47–1.50 Å and 1.34–1.38 Å, respectively; Allen *et al.*, 1987]. This might be indicative of a slight π electron conjugation of the sulphonamide N with the pyridinium ring. The benzene ring exhibits an angle of 109.1 (2) ° with the pyridinium ring. The dihedral angle between the nitro group and the benzene ring is 2.2 (1) °.

The crystal structure is stabilized by O—H···N, O—H···O and N—H···O hydrogen bonds (Table 1).

### Experimental

A solution of 4-nitrobenzenesulfonyl chloride (2.2 g, 10 mmol) in CH_2_Cl_2_ (10 ml) was added dropwise to a suspension of 4-aminopyridine (0.9 g, 10 mmol) in CH_2_Cl_2_ (10 ml) at room temperature with stirring. The reaction mixture was stirred overnight. The yellow solid obtained was washed with warm water to obtain the title compound in a yield of 55.3%. A colourless single-crystal suitable for X-ray analysis was obtained by slow evaporation of an acetic acid solution at room temperature over a period of a week.

### Refinement

The H atoms of the water molecule were found on a difference Fourier map and refined freely. The N-bound H atoms were located in a difference map and their coordinates were refined with *U*_iso_(H) = 1.2*U*_eq_(N). The C-bound H atoms were positioned geometrically (C—H = 0.95 Å) and refined as riding with *U*_iso_(H) = 1.2*U*_eq_(C).

### Crystal data

**Table:**

C_11_H_9_N_3_O_4_S·H_2_O	*F*(000) = 616
*M**_r_* = 297.29	*D*_x_ = 1.600 Mg m^−^^3^
Monoclinic, *P*2_1_/*n*	Mo *K*α radiation, λ = 0.71073 Å
Hall symbol: -P 2yn	Cell parameters from 3340 reflections
*a* = 6.7766 (14) Å	θ = 1.9–27.9°
*b* = 8.3932 (17) Å	µ = 0.29 mm^−^^1^
*c* = 21.717 (4) Å	*T* = 113 K
β = 92.35 (3)°	Block, colourless
*V* = 1234.2 (4) Å^3^	0.20 × 0.18 × 0.12 mm
*Z* = 4	

### Data collection

**Table:**

Rigaku Saturn CCD area-detector diffractometer	2908 independent reflections
Radiation source: rotating anode	2022 reflections with *I* > 2σ(*I*)
confocal	*R*_int_ = 0.061
Detector resolution: 7.31 pixels mm^-1^	θ_max_ = 27.9°, θ_min_ = 1.9°
ω and φ scans	*h* = −8→7
Absorption correction: multi-scan (CrystalClear; Rigaku/MSC, 2005)	*k* = −11→10
*T*_min_ = 0.932, *T*_max_ = 0.963	*l* = −19→28
9708 measured reflections	

### Refinement

**Table:**

Refinement on *F*^2^	Primary atom site location: structure-invariant direct methods
Least-squares matrix: full	Secondary atom site location: difference Fourier map
*R*[*F*^2^ > 2σ(*F*^2^)] = 0.055	Hydrogen site location: inferred from neighbouring sites
*wR*(*F*^2^) = 0.136	H atoms treated by a mixture of independent and constrained refinement
*S* = 1.17	*w* = 1/[σ^2^(*F*_o_^2^) + (0.031*P*)^2^ + 1.5168*P*] where *P* = (*F*_o_^2^ + 2*F*_c_^2^)/3
2908 reflections	(Δ/σ)_max_ = 0.002
193 parameters	Δρ_max_ = 0.35 e Å^−^^3^
0 restraints	Δρ_min_ = −0.45 e Å^−^^3^

### Special details

**Table:**

Geometry. All e.s.d.'s (except the e.s.d. in the dihedral angle between two l.s. planes) are estimated using the full covariance matrix. The cell e.s.d.'s are taken into account individually in the estimation of e.s.d.'s in distances, angles and torsion angles; correlations between e.s.d.'s in cell parameters are only used when they are defined by crystal symmetry. An approximate (isotropic) treatment of cell e.s.d.'s is used for estimating e.s.d.'s involving l.s. planes.
Refinement. Refinement of *F*^2^ against ALL reflections. The weighted *R*-factor *wR* and goodness of fit *S* are based on *F*^2^, conventional *R*-factors *R* are based on *F*, with *F* set to zero for negative *F*^2^. The threshold expression of *F*^2^ > σ(*F*^2^) is used only for calculating *R*-factors(gt) *etc*. and is not relevant to the choice of reflections for refinement. *R*-factors based on *F*^2^ are statistically about twice as large as those based on *F*, and *R*- factors based on ALL data will be even larger.

### Fractional atomic coordinates and isotropic or equivalent isotropic displacement parameters (Å^2^)

**Table:**

	*x*	*y*	*z*	*U*_iso_*/*U*_eq_	
S1	0.89206 (11)	0.18818 (8)	0.12547 (3)	0.01574 (18)	
N1	0.7145 (4)	0.7176 (3)	0.00969 (13)	0.0228 (6)	
H1A	0.707 (6)	0.823 (5)	−0.0019 (19)	0.048 (12)*	
N2	0.8061 (4)	0.2477 (3)	0.06005 (11)	0.0166 (5)	
N3	0.2350 (4)	0.1341 (3)	0.29976 (12)	0.0236 (6)	
O1	1.0378 (3)	0.2937 (2)	0.15368 (9)	0.0197 (5)	
O2	0.9523 (3)	0.0240 (2)	0.11577 (10)	0.0208 (5)	
O3	0.2477 (4)	0.2106 (3)	0.34777 (10)	0.0332 (6)	
O4	0.0934 (4)	0.0497 (3)	0.28506 (13)	0.0401 (7)	
C1	0.7738 (4)	0.4058 (3)	0.04742 (13)	0.0151 (6)	
C2	0.7618 (5)	0.5312 (3)	0.09068 (14)	0.0203 (6)	
H2	0.7736	0.5099	0.1336	0.024*	
C3	0.7329 (5)	0.6835 (4)	0.06978 (16)	0.0259 (7)	
H3	0.7257	0.7677	0.0989	0.031*	
C4	0.7199 (5)	0.6006 (4)	−0.03300 (14)	0.0204 (6)	
H4	0.7053	0.6265	−0.0755	0.024*	
C5	0.7463 (4)	0.4454 (3)	−0.01543 (14)	0.0176 (6)	
H5	0.7462	0.3638	−0.0458	0.021*	
C6	0.6953 (4)	0.1762 (3)	0.17671 (13)	0.0150 (6)	
C7	0.5306 (5)	0.0811 (3)	0.16067 (13)	0.0182 (6)	
H7	0.5230	0.0275	0.1221	0.022*	
C8	0.3798 (5)	0.0659 (3)	0.20114 (14)	0.0180 (6)	
H8	0.2678	0.0013	0.1912	0.022*	
C9	0.3961 (4)	0.1475 (3)	0.25685 (13)	0.0171 (6)	
C10	0.5550 (5)	0.2447 (3)	0.27298 (14)	0.0199 (6)	
H10	0.5598	0.3011	0.3109	0.024*	
C11	0.7072 (5)	0.2580 (3)	0.23239 (13)	0.0189 (6)	
H11	0.8189	0.3226	0.2426	0.023*	
O5	0.7268 (4)	0.0224 (2)	−0.03361 (11)	0.0216 (5)	
H5A	0.763 (6)	0.079 (5)	−0.003 (2)	0.046 (13)*	
H5B	0.815 (7)	0.027 (5)	−0.060 (2)	0.049 (14)*	

### Atomic displacement parameters (Å^2^)

**Table:**

	*U*^11^	*U*^22^	*U*^33^	*U*^12^	*U*^13^	*U*^23^
S1	0.0176 (4)	0.0143 (3)	0.0154 (3)	0.0017 (3)	0.0016 (3)	0.0006 (3)
N1	0.0216 (15)	0.0139 (11)	0.0326 (15)	−0.0023 (10)	−0.0021 (12)	0.0047 (11)
N2	0.0213 (14)	0.0133 (11)	0.0152 (11)	0.0014 (10)	0.0005 (10)	0.0005 (9)
N3	0.0253 (15)	0.0227 (13)	0.0231 (14)	0.0026 (11)	0.0061 (12)	0.0037 (11)
O1	0.0175 (11)	0.0213 (10)	0.0203 (11)	−0.0013 (9)	−0.0007 (8)	−0.0004 (9)
O2	0.0254 (12)	0.0151 (10)	0.0222 (11)	0.0054 (8)	0.0056 (9)	0.0025 (8)
O3	0.0445 (16)	0.0358 (13)	0.0202 (11)	−0.0024 (11)	0.0134 (11)	−0.0026 (10)
O4	0.0294 (15)	0.0468 (15)	0.0452 (16)	−0.0171 (12)	0.0146 (13)	−0.0081 (13)
C1	0.0116 (15)	0.0138 (12)	0.0201 (14)	−0.0010 (10)	0.0029 (12)	0.0012 (11)
C2	0.0251 (17)	0.0172 (13)	0.0184 (14)	0.0024 (12)	−0.0027 (13)	−0.0017 (12)
C3	0.0309 (19)	0.0169 (14)	0.0292 (17)	0.0010 (13)	−0.0068 (14)	−0.0050 (13)
C4	0.0158 (16)	0.0244 (14)	0.0211 (15)	0.0013 (12)	0.0026 (12)	0.0048 (12)
C5	0.0135 (15)	0.0199 (13)	0.0196 (14)	0.0005 (11)	0.0037 (12)	−0.0003 (12)
C6	0.0155 (15)	0.0147 (12)	0.0148 (13)	0.0028 (11)	0.0008 (11)	0.0019 (11)
C7	0.0204 (16)	0.0189 (13)	0.0150 (13)	0.0013 (12)	−0.0024 (12)	−0.0021 (11)
C8	0.0164 (16)	0.0166 (13)	0.0207 (14)	0.0005 (11)	−0.0012 (12)	−0.0003 (12)
C9	0.0180 (15)	0.0170 (13)	0.0165 (14)	0.0022 (11)	0.0033 (12)	0.0033 (11)
C10	0.0241 (17)	0.0205 (13)	0.0151 (13)	0.0021 (12)	0.0000 (12)	−0.0035 (12)
C11	0.0221 (16)	0.0190 (13)	0.0156 (13)	−0.0026 (12)	−0.0008 (12)	−0.0012 (11)
O5	0.0292 (14)	0.0158 (10)	0.0199 (11)	−0.0026 (9)	0.0037 (10)	−0.0011 (9)

### Geometric parameters (Å, °)

**Table:**

S1—O1	1.444 (2)	C4—C5	1.367 (4)
S1—O2	1.455 (2)	C4—H4	0.9500
S1—N2	1.594 (2)	C5—H5	0.9500
S1—C6	1.774 (3)	C6—C11	1.390 (4)
N1—C3	1.337 (4)	C6—C7	1.404 (4)
N1—C4	1.352 (4)	C7—C8	1.381 (4)
N1—H1A	0.92 (4)	C7—H7	0.9500
N2—C1	1.370 (3)	C8—C9	1.391 (4)
N3—O4	1.224 (4)	C8—H8	0.9500
N3—O3	1.225 (3)	C9—C10	1.385 (4)
N3—C9	1.468 (4)	C10—C11	1.388 (4)
C1—C5	1.410 (4)	C10—H10	0.9500
C1—C2	1.416 (4)	C11—H11	0.9500
C2—C3	1.368 (4)	O5—H5A	0.85 (4)
C2—H2	0.9500	O5—H5B	0.85 (5)
C3—H3	0.9500		
			
O1—S1—O2	116.81 (13)	C5—C4—H4	119.8
O1—S1—N2	113.86 (13)	C4—C5—C1	120.4 (3)
O2—S1—N2	105.21 (12)	C4—C5—H5	119.8
O1—S1—C6	106.69 (13)	C1—C5—H5	119.8
O2—S1—C6	105.05 (13)	C11—C6—C7	120.9 (3)
N2—S1—C6	108.69 (13)	C11—C6—S1	120.0 (2)
C3—N1—C4	120.7 (3)	C7—C6—S1	119.1 (2)
C3—N1—H1A	118 (3)	C8—C7—C6	119.8 (3)
C4—N1—H1A	121 (3)	C8—C7—H7	120.1
C1—N2—S1	122.1 (2)	C6—C7—H7	120.1
O4—N3—O3	123.5 (3)	C7—C8—C9	118.2 (3)
O4—N3—C9	118.3 (3)	C7—C8—H8	120.9
O3—N3—C9	118.2 (3)	C9—C8—H8	120.9
N2—C1—C5	115.9 (2)	C10—C9—C8	122.9 (3)
N2—C1—C2	126.9 (3)	C10—C9—N3	118.4 (3)
C5—C1—C2	117.2 (3)	C8—C9—N3	118.7 (3)
C3—C2—C1	119.1 (3)	C9—C10—C11	118.5 (3)
C3—C2—H2	120.5	C9—C10—H10	120.8
C1—C2—H2	120.5	C11—C10—H10	120.8
N1—C3—C2	122.0 (3)	C10—C11—C6	119.6 (3)
N1—C3—H3	119.0	C10—C11—H11	120.2
C2—C3—H3	119.0	C6—C11—H11	120.2
N1—C4—C5	120.5 (3)	H5A—O5—H5B	109 (4)
N1—C4—H4	119.8		
			
O1—S1—N2—C1	−36.5 (3)	O2—S1—C6—C7	−55.7 (3)
O2—S1—N2—C1	−165.6 (2)	N2—S1—C6—C7	56.5 (3)
C6—S1—N2—C1	82.3 (3)	C11—C6—C7—C8	−1.3 (4)
S1—N2—C1—C5	164.4 (2)	S1—C6—C7—C8	177.6 (2)
S1—N2—C1—C2	−16.8 (4)	C6—C7—C8—C9	0.5 (4)
N2—C1—C2—C3	178.6 (3)	C7—C8—C9—C10	1.0 (4)
C5—C1—C2—C3	−2.7 (5)	C7—C8—C9—N3	179.0 (3)
C4—N1—C3—C2	1.4 (5)	O4—N3—C9—C10	179.1 (3)
C1—C2—C3—N1	0.4 (5)	O3—N3—C9—C10	0.6 (4)
C3—N1—C4—C5	−0.6 (5)	O4—N3—C9—C8	0.9 (4)
N1—C4—C5—C1	−1.8 (5)	O3—N3—C9—C8	−177.6 (3)
N2—C1—C5—C4	−177.7 (3)	C8—C9—C10—C11	−1.7 (4)
C2—C1—C5—C4	3.4 (4)	N3—C9—C10—C11	−179.8 (3)
O1—S1—C6—C11	−1.5 (3)	C9—C10—C11—C6	1.0 (4)
O2—S1—C6—C11	123.1 (2)	C7—C6—C11—C10	0.5 (4)
N2—S1—C6—C11	−124.7 (2)	S1—C6—C11—C10	−178.3 (2)
O1—S1—C6—C7	179.7 (2)		

### Hydrogen-bond geometry (Å, °)

**Table:**

*D*—H···*A*	*D*—H	H···*A*	*D*···*A*	*D*—H···*A*
O5—H5A···N2	0.85 (4)	1.97 (4)	2.813 (3)	167 (4)
O5—H5B···O2^i^	0.85 (5)	2.07 (5)	2.895 (3)	164 (4)
N1—H1A···O5^ii^	0.92 (4)	1.82 (4)	2.728 (3)	170 (4)

Symmetry codes: (i) −*x*+2, −*y*, −*z*; (ii) *x*, *y*+1, *z*.
